# Germ cell specification and pluripotency in mammals: a perspective from early embryogenesis

**DOI:** 10.1007/s12522-014-0184-2

**Published:** 2014-06-10

**Authors:** Naoko Irie, Walfred W. C. Tang, M. Azim Surani

**Affiliations:** ^1^ Wellcome Trust/Cancer Research UK, Gurdon Institute University of Cambridge Tennis Court Road CB2 1QN Cambridge UK

**Keywords:** Epiblast, Human, Mouse, Pluripotent stem cells, Primordial germ cells

## Abstract

Germ cells are unique cell types that generate a totipotent zygote upon fertilization, giving rise to the next generation in mammals and many other multicellular organisms. How germ cells acquire this ability has been of considerable interest. In mammals, primordial germ cells (PGCs), the precursors of sperm and oocytes, are specified around the time of gastrulation. PGCs are induced by signals from the surrounding extra‐embryonic tissues to the equipotent epiblast cells that give rise to all cell types. Currently, the mechanism of PGC specification in mammals is best understood from studies in mice. Following implantation, the epiblast cells develop as an egg cylinder while the extra‐embryonic ectoderm cells which are the source of important signals for PGC specification are located over the egg cylinder. However, in most cases, including humans, the epiblast cells develop as a planar disc, which alters the organization and the source of the signaling for cell fates. This, in turn, might have an effect on the precise mechanism of PGC specification in vivo as well as in vitro using pluripotent embryonic stem cells. Here, we discuss how the key early embryonic differences between rodents and other mammals may affect the establishment of the pluripotency network in vivo and in vitro, and consequently the basis for PGC specification, particularly from pluripotent embryonic stem cells in vitro.

## Introduction

In mammals, germ cells are specified at a very early stage of development from the post‐implantation epiblast cells following blastocyst implantation. The inner cell mass (ICM) of blastocysts is the source of epiblast cells as well as embryonic stem cells (ESCs). The ICM is segregated into epiblast and hypoblast or the primitive endoderm. Epiblast cells are equipotent and give rise to all the somatic cells and germ cells [1], as well as epiblast stem cells (EpiSCs) in vitro. In mice, precursors of the primordial germ cells (PGCs) are specified in the extreme proximal region of the epiblast adjacent to the extra‐embryonic ectoderm (ExE) [2, 3]. Subsequently, nascent PGCs proliferate and migrate through the developing hindgut into the genital ridges [4]. PGCs stain strongly and specifically for alkaline phosphatase (AP) [5, 6, 7]. PGCs are also able to become pluripotent stem cells (PSCs) in vitro, called embryonic germ cells (EGCs) under defined culture conditions [8, 9].

Many studies on mammalian development and PGC specification have been conducted in the mouse model. However, there are some key embryological differences between mice and other mammals, especially at the epiblast stage when PGCs are specified. For example, rodent epiblast forms a cup‐shaped egg cylinder but most other mammals have a flat disc‐like epiblast. Signals from extra‐embryonic tissues induce germ cell fate in a subset of epiblast at a specific position with optimal concentration and timing of the signals. As PGC specification largely depends on signals from surrounding tissues, the morphology of the embryo is a crucial consideration for dissecting the mechanism of germline establishment in different mammals. Earlier events, such as formation of epiblast from zygotes as well as establishment of pluripotency, are also fundamental for PGC specification, since PGCs share some key features with pluripotent cells in vivo and in vitro. Thus, differences during early embryogenesis among mammals are essential to understanding the development of mammalian germ cells.

There have been successful attempts to recapitulate germ cell specification in vitro using mouse PSCs, but no similar or extensive studies have been described in other mammals. It is possible that differences in PSCs and pluripotency signaling between rodents and other mammals may reflect differences in their early embryology, and therefore the underlying mechanism of germ cell specification. By appreciating these fundamental discrepancies, we propose strategies to further dissect the mechanism of human germ cell specification and the pluripotency network.

## Pre‐implantation embryogenesis and pluripotency in mammals

There are differences between rodents and the other mammals as early as zygote formation. The centrosome, which is critical for successful fertilization, is contributed by sperm in most mammals, but by oocytes in rodents [10]. Global DNA demethylation in early embryos for active paternal DNA demethylation in zygotes is known to occur in mice and rats [11], but only partially in humans and rabbits [12, 13]. X chromosome inactivation in female mouse embryos first occurs in response to the paternal imprint of *Xist* non‐coding RNA transcript at the 2‐ to 4‐cell stage followed by paternal X chromosome inactivation [14], which persists in the extra‐embryonic tissues. However, in the embryo, paternal X chromosome reactivation precedes random X inactivation in the ICM [15]. In contrast, transcripts of *Xist* are detected from both X chromosomes in human and rabbit early embryos [15, 16, 17, 18]. In rabbits, *Xist* expression becomes monoallelic only at the late blastocyst stage, first in the trophoblast, and then in the embryonic cells. The functional consequence of *Xist* expression, i.e., repression of X‐linked genes, seems to occur only at the blastocyst stage in rabbits [15]. Both the non‐imprinted early biallelic expression of *Xist* and the delay of X‐linked genes inactivation are common to rabbit and human embryos. Thus, the mouse appears to show unique DNA demethylation and X chromosome inactivation mechanisms compared to humans and rabbits.

**Figure 1:**
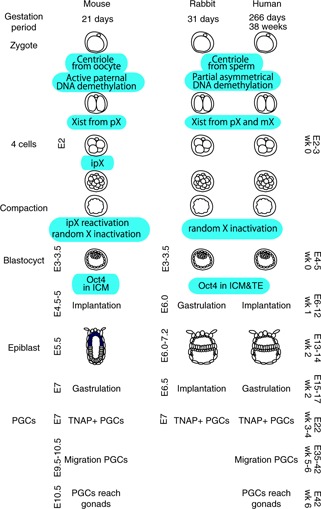
Comparison of early embryogenesis of mice, rabbits and humans from zygote to epiblast stage and during PGC differentiation. *pX* paternal X chromosome, *mX* maternal X chromosome, *ipX* inactivated paternal X chromosome, *ICM* inner cell mass, *TE* trophectoderm, *PGC* primordial germ cell, *TNAP* tissue‐nonspecific alkaline phosphatase (AP)

After trophectoderm (TE) and ICM formation at the blastocyst stage, the embryo undergoes remethylation of DNA. In humans, 5‐methylcytosine is higher in the TE than in the ICM while in the mouse it is the other way round [12]. On the other hand, both ICM and TE DNA in bovine blastocysts are highly methylated. Early cell lineage commitment during blastocyst formation is another example where the embryos of different mammals clearly vary between species [19] (Fig. 1).

## Regulation of pluripotency molecules in pre‐implantation embryos in mammals

OCT4, the octamer‐binding transcription factor (also known as POU5F1) is essential for the establishment of pluripotency during early embryogenesis and in in vitro PSCs.

In mice, Oct4 and Cdx2 are essential for formation of ICM and TE, respectively [20, 21, 22]. Cdx2 represses Oct4 in mouse TE of early blastocysts, but in humans, rabbits, cows and some other mammals, OCT4 expression persists in the TE until the late blastocyst stage [23, 24, 25, 26, 27, 28, 29, 30, 31, 32, 33, 34]. In bovines, Cdx2 is required for TE maintenance but not for repression of Oct4 expression. Interestingly, mouse Oct4 promoter has Tcfap2 (required for trophectoderm maintenance and PGC development in mice) binding sites mediating Oct4 repression. However, bovine, human, and rabbit Oct4 promoters do not contain these sites and maintain high Oct4 levels in the TE [24]. Indeed, early TE cells from bovine embryos can contribute to chimeric embryos after introduction to blastocysts [19]. Furthermore, the plating of intact human blastocysts resulted predominantly in the outgrowth of TE‐like cells, rather than leading to ESC derivation as in the case of mice [35]. This suggests that regulation of pluripotency in early embryos seems to be different in mice compared to other mammals (Fig. 1).

## Gastrulation‐stage/peri‐implantation embryo and primordial germ cell specification in mammals

In mammals, the body plan is set with regard to axis formation and the starting point for germ layer formation during gastrulation. One of the critical events at this stage is PGC specification in the epiblast.

**Figure 2:**
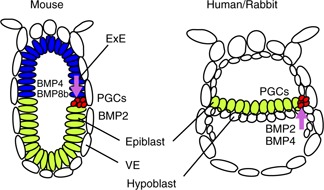
Primordial germ cell specification of mice, humans and rabbits is induced from signals such as BMPs from surrounding tissues at pre‐implantation epiblast stage. Mouse epiblast is an egg cylinder and human/rabbit epiblast is a flat disc‐shaped epiblast. *ExE* extraembryonic ectoderm, *VE* visceral endoderm

There are topological differences with respect to the arrangement and the timing involved of the start of gastrulation and implantation [36] (Fig. 2). While a mouse blastocyst implants in the uterus by E4.5, a human blastocyst grows for a little longer before implanting at E6–12 with highly invasive trophoblast outgrowth ahead of gastrulation. In rabbits, cows, pigs and sheep, blastocysts undergo gastrulation prior to implantation [19, 37]. Cow embryo implantation occurs particularly late, i.e., >5 days following germ layer formation and 10 days after blastocyst formation [38]. However, the pattern of *brachyury* gene expression which is a marker of vertebrate gastrulation in the bovine embryo is similar to the pattern found in mice [39]. These observations suggest that gastrulation in mammals is regulated irrespective of implantation [40]. However, the schedule of gastrulation and implantation has a considerable effect on the size and mutual contact areas of the trophoblast, epiblast and hypoblast of mammalian embryos.

The embryo proper of most gastrulation‐stage mammals, including humans, rabbits and pigs, has the shape of a flat disc with two cell layers—epiblast and hypoblast (Figs. 1, 2) [37, 41, 42, 43]. However, in rodents, the embryonic disc is forced into a complex shape called the ‘egg cylinder’ in which the anterior and posterior poles of the embryo come to lie in close proximity to each other, whereby an additional proximal–distal body axis has to be taken into account (Fig. 2) [44].

In mice, when the syncytiotrophoblast starts to penetrate the wall of the uterus, the epiblast and hypoblast are physically constrained and form a bilaminar embryo within 12 h [45]. The internal epiblast cells reorganize from a ball of cells into a cup‐shaped epithelium surrounded by hypoblast. Immediately before gastrulation (E6.0 and E6.5), the mouse embryo can be visualized as a thick‐walled cup of tissue (the epiblast or embryonic ectoderm), which gives rise to the entire fetus and some of the placental membranes. A second thick‐walled cup of tissue (the ExE) placed overturned on the epiblast will give rise to the main part of the placenta. Both cups are enclosed in a thin bag of primitive endoderm. Around E4.5 and E5.5, the ExE arises from the polar TE and makes contact with the underlying epiblast, which expresses BMP4, a critical factor for PGC specification. At E6.5, gastrulation starts with the formation of the primitive streak at the posterior region of the embryo. Epiblast cells migrating first through this structure include the PGC precursors which form the extra‐embryonic mesoderm.

In humans, the formation of the embryonic bilaminar disc occurs after implantation and prior to embryonic folding (between about E14–21). The embryonic disc is derived from the epiblast layer, which lies between the hypoblast layer and the amnion and is derived from the ICM. The formation of the bilaminar embryonic disc precedes gastrulation. Following gastrulation, polar TE above the epiblast differentiates into the syncytiotrophoblast that invades the uterine tissue and the cytotrophoblast contacting the epiblast. At the beginning of the third week, the primitive streak appears and the gastrulation begins. The hypoblast in human can be considered equivalent to the mouse visceral endoderm (VE), while no structure equivalent to the mouse ExE apparently exists. Around the end of the third week, the place where PGCs can be first identified in human embryos is the same as in the mouse, i.e., in the endoderm of the wall of the yolk sac at an angle with the allantois [46].

Gastrulation in the rabbit starts at E6, i.e., at a stage when implantation has not yet started. A crescent‐like dense area in the anterior part of the embryonic disc appears [47], followed by a sickle‐shaped elongation of reduced density at the posterior pole (posterior gastrula extension, PGE) about 6 h later [48]. The primitive streak appears in the midline of the PGE generating the first mesoderm cells. The mesoderm is formed by epithelio‐mesenchymal transition of epiblast cells under the ‘fine‐tuning’ influence of the hypoblast [49]. The movement, migration, and epithelio‐mesenchymal transformation of epiblast cells result in the formation of the primitive streak [48] until it encompasses up to half of the longitudinal axis of the embryonic disc. The appearance of Hensen's node at the tip of the primitive streak coincides with the time when implantation starts.

One of the most important events—PGC induction in epiblast cells—occurs at this stage, and is dependent on signals from surrounding tissue. The most critical structure in mice for PGC specification—ExE secreting BMP4— does not exist as the same structure in the other mammals. These differences may have a critical effect on PGC specification factors.

## Germ cell lineage specification in vivo

PGCs arise at the onset of gastrulation through a process of inductive signaling. Specific signals secreted by neighboring cells induce the commitment and specification of PGC precursors in a subset of epiblast cells. Specified PGCs migrate from an extra‐embryonic region into the embryo proper, then move through the hindgut and dorsal mesentery into the developing genital ridges, where they undergo sexual differentiation. Concomitant to migration, PGCs undergo comprehensive epigenetic reprogramming, which includes imprint erasure, X‐reactivation, global DNA demethylation and dynamic changes in histone modification states.

In mice, signals from ExE and VE play an essential role in the induction of PGCs. BMP signaling is indispensable for mouse PGC specification. Mutant embryos with targeted disruption of BMP signaling components, including *Bmp2*, *Bmp4*, *Bmp8b*, *Smad1*, *Smad4*, *Smad5* or *Alk2*, all demonstrated loss or reduced numbers of AP‐positive (AP+) PGCs. Blimp1 (B‐lymphocyte‐induced maturation protein 1, also known as Prdm1) is the earliest known marker of nascent PGCs [50]. BMP4 and BMP8b secreted by the ExE and BMP2 from the proximal VE induce the formation of Blimp1‐positive (+) PGC precursors at the posterior proximal epiblast in the pregastrulation embryo at E6.25 (Fig. 2). Induction of Blimp1+ PGC precursors in isolated E6 epiblast relies on BMP4 and BMP2 in a dose‐dependent manner, from which BMP4 is the most potent inducer. Specified PGCs are restricted to the posterior epiblast, apparently due to antagonistic signals emitted from the anterior VE that is adjacent to the anterior epiblast. These inhibitory signals, which include Cer1 against BMP and Nodal, Lefty1 against Nodal, and Dkk1 against Wnt, prevent posteriorization of the anterior epiblast. Interestingly, *Smad2* and *FoxH1* mutant embryos, which lack the anterior VE, showed Blimp1+ PGC induction in both anterior and posterior proximal epiblast [51]. WNT signaling has also been implicated in PGC specification. *Wnt3* is initially expressed in both the anterior and posterior epiblast of the egg cylinder at E6.25; it is then restricted to the posterior proximal epiblast and the proximal VE [52]. *Wnt3* knockout embryos develop a normal egg cylinder but do not form a primitive streak and mesoderm. Blimp1+ PGCs are absent in Wnt3 mutant embryos at E7.5 [51]. Although *Wnt3*‐deficient embryos emit BMP4 from ExE and express BMP signaling components, the epiblast of these mutants failed to respond to BMP4 and showed the absence of phosphorylated Smad1/5/8 (indicator of active BMP signaling). Thus, Wnt3 may be necessary for the epiblast to achieve competence to respond to BMP signaling for germ cell formation. Interestingly, WNT3 induces many transcription factors associated with mesoderm in in vitro epiblast‐like cells (EpiLCs) through β‐catenin. Among these, T (also known as brachyury) was essential for robust activation of Blimp1 and Prdm14 by binding distinct regulatory elements of both Blimp1 and Prdm14 genes directly. WNT3 has a permissive role of BMP4 in PGC specification [53].

Signaling pathways/networks for PGC specification in vivo in other mammals, including humans, are largely unexplored. Next to mice, rabbits are the only other mammal in which BMP signaling and PGC specification has been studied [54] (Fig. 2). In order to relate the two distinct configurations, Behringer et al. [55] proposed a flattened model of the mouse embryo. While the hypoblast underneath the epiblast in the embryonic disc may be equivalent to VE in mice, the extra‐embryonic trophoblast and yolk sac epithelium immediately surrounding the periphery of the embryonic disc can be regarded as rodent ExE and extra‐embryonic VE, respectively. Interestingly, in rabbits (a flat disc‐like epiblast), *BMP2* and *BMP4* are enriched in annular domains at the boundary of the embryonic disc, which corresponds to the junction between the proximal epiblast, the ExE and the surrounding VE in mice, where PGCs are specified from (Fig. 2) [54]. In pregastrulation rabbit embryos, *BMP2* is first expressed from the hypoblast and yolk sac epithelium at the boundary of the embryonic disc, which is equivalent to the proximal VE and extra‐embryonic VE in mice, respectively. Rabbit *BMP4* expression is significantly delayed compared to the mouse. In rabbits, *BMP4* is first detected during primitive streak formation and is expressed peripherally in intra‐embryonic hypoblast and epiblast and in the mesoderm at the posterior pole of the embryonic disc. Interestingly, *Blimp1*+ single PGC precursors are detected before primitive streak formation and *Blimp1* mRNA distribution closely follows the expression pattern of *BMP2*. Thus, BMP2 may play a more essential role in rabbit PGC specification than BMP4. Regarding antagonistic signals, mRNA of *Cer1* is restricted to the anterior region of the embryonic disc as well as the anterior primitive streak in rabbits [49]. This is likely to restrict PGC specification to the posterior epiblast. Further expression studies are necessary to reveal the potential roles of BMPs and other signals, such as Wnt3 and BMP8b, in non‐rodent PGC specification.

In mice, shortly after the induction of Blimp1, PGC precursors begin to express another two key transcription factors, Prdm14 (PR domain‐containing protein 14) and Tcfap2c (transcription factor AP‐2, gamma), at E6.5 and E6.75, respectively. As PGCs are specified from posterior epiblast cells originally primed towards a somatic fate, nascent PGCs initially express mesodermal genes such as Hoxa1, Hoxb1 and T. However, Blimp1, Prdm14 and Tcfap2c form a tripartite transcription factor network which facilitates mouse PGC specification by suppressing somatic gene expression, initiating the germ cell transcriptional program, and triggering genome‐wide epigenetic reprogramming [56]. Knockout embryos of any of the three factors lose early germ cells due to failure of the early PGC specification processes. In contrast, overexpression of these three factors together in competent EpiLCs derived in vitro (see later sections) is sufficient to induce mouse germ cell formation in the absence of cytokines [56]. This study highlights the essential roles of the three transcription factors in germ cell formation and maintenance. With the establishment of germ cell fate, mouse PGCs increase in number and move out of the embryo through the forming primitive streak to the extra‐embryonic mesoderm at the base of the allantois at E7.25. PGCs form a cluster of cells, which have strong AP activity. From E8 to E11, PGCs migrate into the midgut and hindgut endoderm through the dorsal mesentery, to the forming genital ridge.

In addition to germ cell‐specific genes, such as AP, Nanos3, Dazl, Mvh and Dnd1, mouse PGCs also express pluripotency‐associated genes, including Oct4, Nanog, Sox2, Klf2 and Stella. While *Klf2* (germline phenotype not described in knockout embryos) and *Stella* are apparently dispensable for PGC development [57], the three core pluripotency factors Oct4, Nanog and Sox2 are important for the germline. Oct4 is uniformly expressed in post‐implantation epiblast and also in nascent PGCs during specification. Oct4 expression remains high until germ cells undergo sexual differentiation in the gonad [58, 59]. Oct4 is apparently essential for both germ cell specification [60] and maintenance [61]. Nanog is enriched at the proximal posterior epiblast, the position where PGCs are specified from, in E6.5 and E7.5 embryos [62, 63, 64]. Intriguingly, Stella+ PGCs located proximal to the allantoic rudiment do not show Nanog staining at E7.5, but become positive at E7.75 [63, 65]. It is not clear whether PGCs are specified from Nanog‐negative cells or from Nanog‐positive proximal posterior epiblast which transiently downregulate Nanog after specification. *Nanog*‐null ESCs can contribute to PGCs in chimeric embryos, but these PGCs are lost by E12.5 [66], likely due to apoptosis [67]. Thus, Nanog appears to be dispensable for mouse PGC specification but is essential for germ cell maintenance. Sox2 is detected in mouse PGC from E7.5 onwards. Conditional knockout of Sox2 shortly after specification caused a dramatic decrease of germ cell numbers by E7.5 and are undetectable by E13.5 [68]. Sox2 directly regulates *Kit* expression, which is important for PGC survival and proliferation.

Among the genes critical for mouse PGC specification, *Blimp1*+ PGC precursors were first observed as single epiblast cells in rabbit at the posterior end of the embryonic disc shortly before gastrulation [54]. *Blimp1*+ cells are then observed in the mesoderm at the posterior end of the primitive streak. They are later distributed within a bi‐lobbed area that flanks the posterior margin, where positive staining by germ cell‐specific antibody PG‐2 is also observed [54, 69]. In pig pregastrulation embryos at around E13, Oct4 is expressed uniformly in most of the epiblast, while Nanog is localized to a minor portion of epiblast, which are scattered throughout the embryonic disc and have Oct4 downregulated. Interestingly, at the same stage, some of the marginal posterior epiblast cells co‐express OCT4 and NANOG and are likely to be PGC precursors. After formation of the primitive streak at E15, Nanog is clearly restricted to OCT4+ PGCs at the posterior pole of the epiblast. Similar to mouse, these pig PGC precursors later form a cluster at the posterior end of the filamentous embryo and can also be found as individual cells at the wall of the yolk sac. Thus, OCT4 and NANOG are both expressed during pig PGC specification and may play a part in the process. While the expression of NANOG at subsequent stages is unknown, pig PGCs continue to express OCT4 during migration through the hindgut (E17) and colonization of the genital ridges at E20 until at least E28 [70]. Migratory pig PGCs also express other germ cell markers, including AP, cKIT, SSEA1 and EMA1 [71]. In addition to pig, OCT4 has also been reported to be expressed in canine and sheep PGCs [72, 73].

Due to ethical and technical reasons, there is little information on the origin of human PGCs in post‐implantation embryos. In later stages, human PGCs are distinguished by their large size, spherical shape, the presence of abundant glycogen granules in the cytoplasm and prominent nucleoli. Human PGCs are first identified at E24 in the extra‐embryonic yolk sac close to the junction with the allantois [74], similar to the position of mouse PGCs at E8. A few days later, at E26, PGCs are found in the hindgut and they migrate into the dorsal mesentery at E28. By E37, human PGCs have colonized the genital ridges. In general, human migratory PGCs/gonocytes express a similar set of markers to mouse PGCs, including BLIMP1, TFAP2C, OCT4, NANOG, AP, SSEA1, cKIT, VASA and DAZL. While Sox2 has been shown to be essential for early mouse PGC development, it is surprisingly not expressed in human PGCs. Instead of SOX2, another SOX family member SOX17 is found in human PGCs [75]. The expression of other key mouse PGCs markers, such as Prdm14 and Stella, remain to be investigated.

## In vitro PSCs from mammals—ESCs, EpiSCs, EGCs and iPSCs

**Figure 3:**
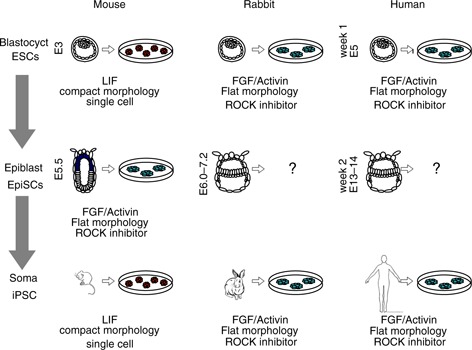
PSCs from various developmental stages of mice, rabbits and humans. There are two major types of PSCs—the naive state which are dependent on LIF and have compact colonies and the primed state which are dependent on FGF and activin and have flat colonies. Naive PSCs are able to be dissociated and passaged as single cells, while primed PSCs are passaged as a small clump of cells or with ROCK inhibitors

Pluripotent cell lines have now been established from a variety of mammals. There are both similarities and differences in the morphology of the colonies and the signaling and transcriptional regulation for maintaining the pluripotency of mammalian stem cells (Fig. 3).

The first ESC line was established from mouse blastocyst in 1981 [76, 77], followed by primates (rhesus macaques) in 1995 [78], marmosets in 1996 [79], and finally humans in 1998 [80]. Furthermore, ESCs or ES‐like cells have been derived from the rabbit [81, 82, 83], and pig [84]; however, in the majority of studies the ESCs and induced PSCs (iPSCs), besides mice and humans, did not meet all criteria for pluripotency, specifically in the in vivo tests.

On the other hand, EpiSCs are derived from mouse post‐implantation embryo [85, 86] and presumptive EpiSCs are derived from pig [84]. EGCs are established from mouse PGCs [8, 9] and have been attempted from human [46], rabbit [87], pig [84] and cattle [84] PGCs. After the discovery of iPSCs from mouse cells [88], this was followed by human [89], monkey [90], rabbit [91], and pig [84].

Most of the in vitro PSCs grow as AP+ colonies and share the expression of pluripotent regulatory genes, OCT4, SOX2, and NANOG. Stem cells in vitro can generally be divided into two types by regulatory signaling and morphology. One is dependent on leukemia inhibitor factor (LIF) and forms a compact dome‐shaped colony, and the other is not dependent on LIF but sometimes dependent on FGF2, forming larger flattened colonies, which cannot be passaged as single cells. They are termed ‘naive’ and ‘primed’, respectively, although there are some PSC lines that show both or intermediate features of naive and primed (Fig. 3). In the case of mouse cells, ESCs are from blastocysts and EpiSCs are from post‐implantation epiblast; both express Oct4 but are driven by different enhancers [86]. Interestingly, most ESCs apart from rodents, share defining features of ‘primed’ PSCs. iPSCs from somatic cells in mice have naive characteristics, while human iPSCs have primed characteristics. Mouse EpiSCs are often compared to human ESCs as they largely conform to the ‘primed’ state, although their transcriptional network has some key differences. For example, critical pluripotent genes PRDM14, REX1 and STELLA, which are expressed in human ESCs/iPSC, are not expressed in mouse EpiSCs [92, 93].

Mouse ESCs/iPSCs require LIF, which activates the Jak/Stat3 pathway, and BMP4, which is part of the transforming growth factor‐beta (TGF‐ß) signaling pathway and promotes the expression of inhibitors of differentiation. The Wnt signaling pathway has also been implicated in maintaining mouse ESC self‐renewal and the naive pluripotent state [94]. The addition of Wnt and LIF in a defined condition is sufficient to support mouse ESC self‐renewal. Self‐renewal of mouse EGCs requires LIF‐STAT3 signaling, but LIF signaling is not required for germ cell differentiation. On the other hand, LIF and its related cytokines fail to support human and non‐human primate ESCs in serum‐containing media that supports mouse ESCs [80, 95, 96, 97]. Components of the BMP pathway are all present in human ESCs [98], but unlike mouse ESCs, the addition of BMPs otherwise supports self‐renewal, causes rapid differentiation [99]. Furthermore, WNT/β‐catenin signaling induces human ESC differentiation under chemically defined conditions [100]. Human ESCs/iPSCs require FGF2 and Activin/Nodal supplementation for the derivation and culture of human ESCs. Interestingly, mouse EpiSCs can also be maintained by FGF2 and Activin/Nodal‐supplemented medium.

The Activin/Nodal signaling pathway is necessary for Nanog expression in both mouse EpiSCs and human ESCs [101]. However, while FGF2 is necessary to support human ESCs/iPSCs, it fails to actively support self‐renewal in mouse EpiSCs via Nanog expression. Additionally, in human ESCs, OCT4 binds to the FGF2 promoter establishing an autocrine loop, whereas in mouse EpiSCs, there is no evidence for the regulation of *Fgf2* by Oct4 [101]. On the contrary, FGF2 induces mouse ESCs to differentiate toward the mesodermal lineage. Inhibition of FGF2/ERK signaling by chemical MEK inhibitor plus GSK3 inhibitor shields mouse ESCs from differentiation‐inducing stimuli in a defined condition in the presence of LIF in a naive ‘ground state’ [102].

ES‐like cells and iPSCs from monkeys, rabbits and pigs show flatter colonies (primed state) that resemble human ESCs and mouse EpiSCs but not mouse ESCs. LIF and its related pathways are dispensable for maintenance of undifferentiated status in primate, rabbit, pig ESCs [103, 104]. Treatment of rabbit ESCs with Rho‐associated kinase (ROCK) inhibitor, Y27632, significantly enhanced cell growth similar to human ESCs [91, 105, 106]. Although there is little effect of FGF2 addition on the growth of monkey ESCs [107], FGF2 and Activin/Nodal signaling can maintain the undifferentiated status through Smad2/3 activation of rabbit and porcine ESCs and iPSCs [103, 105]. Interestingly, canine iPSCs are dependent on both FGF2 and LIF in order to maintain their pluripotency [108].

Interestingly, mouse ESCs/iPSCs, EGCs and EpiSCs express SSEA1 as a cell surface marker, while human ESCs/iPSCs express SSEA3, SSEA4, TRA1‐60, TRA‐1‐81 but not SSEA1. On the other hand, human PGCs are known to express SSEA1 [109] and human EGCs also express SSEA1 in addition to SSEA3, SSEA4 and TRA‐1‐60, unlike human ESCs/iPSCs [109, 110]. Human ESCs express the ICM‐associated marker REX1, like naive mouse ESCs, which is not the case in EpiSCs. Human ESCs do not express FGF5, a key EpiSC‐associated marker. Non‐human primate and pig ESCs/iPSCs express SSEA‐3, SSEA‐4, TRA‐1‐60, and TRA‐1‐81 instead of SSEA1, which is similar to human ESCs but not mouse ESCs/iPSCs [103, 107, 111, 112]. Canine PSCs express SSEA1, SSEA4, TRA1‐60, TRA1‐81, and Rex1 [113]. In rabbit ESCs, SSEA‐1, SSEA‐3, SSEA‐4, TRA‐1–60 and TRA‐1–81 are not detectable [81, 82, 91].

While naive mouse ESCs/iPSCs show two active X chromosomes, primed human and pig ESCs/iPSCs and mouse EpiSCs show X chromosome inactivation in females. Canine iPSCs show reactivation of the inactive X chromosome. Interestingly, unlike mouse EpiSCs, primed pig iPSCs can give rise to chimeras with apparent high efficiency [114].

Reversion of primed pluripotent to naive state that can grow in LIF with 2i condition has been attempted [115]. Studies on mouse EpiSCs and human ESCs/iPSCs have included forced expression or addition of extra factors such as Prdm14/Klf2 for mouse EpiSCs [116] and OCT4, KLF2, KLF4 [117], Rarg (RAR‐gamma) and Lrh‐1 (liver receptor homolog 1; Nr5a2) [118] and histone deacetylase inhibitors [119]. Recently, naive‐like human PSCs cultured with a combination of small molecules in addition to 2i and LIF have been reported [120, 121]. Furthermore, naive‐like rabbit iPSCs and pig iPSCs have been reported [122, 123].

Looking through all the reported PSCs, rodent stem cells have some unique features in terms of morphology, signaling and gene expression markers. Therefore, the question arises as to whether or not ‘naive’ state PSCs described for mice exist naturally in other mammals. When comparing mouse ICM and naive ESCs with human ICM and primed ESCs, some common as well as different features are seen [124, 125]. It is hard to conclude that primed human ESCs are not the real pluripotent state for human cells. Another possibility is that because of the differences between mice and the rest of early mammalian embryogenesis, the transient state of naive pluripotency cannot be captured in vitro in the latter. During formation of the rodent egg cylinder, the epiblast cells must reorganize from a ball of cells into cup‐shaped epithelium surrounded by hypoblast. Conversely, in non‐rodent embryo cultures, there may not be a major barrier for progression to primed epiblast, and the opportunity for capturing the transient naive state (if it exists) may be minimal. In another example, monkey blastomeres, but not ICM cells, were shown to generate chimeric monkeys through embryo aggregation, whereas in rodents, both blastomeres and ICM cells have the unrestricted developmental potency to contribute to chimeric animals. This suggests that the state of pluripotency in ICM from non‐rodent mammals may be waning compared to blastomeres.

To understand PSC biology in vitro, even though it might diverge from pluripotent cells in vivo, might provide insights on their differentiation potential, including germ cell biology (Fig. 3).

## In vitro germ lineage differentiation

The ability to generate PGCs from epiblast cells provides the knowledge for the generation of functional PGCs from PSCs in vitro [126], most successfully using mouse PSCs. One of the most defined and efficient protocols of PGC‐like cell (PGC‐LC) induction is from naive mouse ESCs to induce into EpiLCs first by treatment with ActivinA, FGF2, and a low concentration of KSR [127]. The EpiLCs are a transient entity and show a global gene expression profile similar to that of the pre‐gastrulating epiblast at E5.75, but distinct from that of EpiSCs [85, 86, 127]. EpiLCs produce Blimp1, Prdm14, and Stella‐positive PGC‐LCs in the presence of BMP4 and the other cytokines, whereas EpiSCs show some Blimp1 but not Stella expression. The PGC‐LCs show a global gene expression profile very similar to that of PGCs at E9.5, genome‐wide epigenetic reprogramming (reduction of H3K9me2 and elevation of H3K27me3), and undergo spermatogenesis when transplanted into the testes of neonatal W/Wv mice, and the resultant sperm contribute to healthy, fertile offspring [127, 128]. As an alternative strategy, they ectopically induce some key transcription factors for PGC development, such as Blimp1, Prdm14 and Tcfapc2 in EpiLCs and also efficiently induce PGC‐LCs even without cytokine addition. These transcription factor‐induced PGC‐LCs, when transplanted into seminiferous tubules of neonatal mice, can also undergo spermatogenesis and contribute to fertile offspring [129].

Mouse ESCs induced to form PGC‐LCs using spontaneous differentiation protocol exhibit very low efficiency. On the other hand, the defined induction protocol of human PGC‐LCs from human PSCs in vitro has not yet been reported. However, a number of studies show that human PSCs can spontaneously differentiate into PGC‐LCs at a low frequency (around 5 %). The efficiency of spontaneous differentiation to PGCs can be increased with the addition of BMP4, 7, and 8b. Small changes in stem cell culture conditions or co‐culture with human fetal gonad stromal cells, or MEF in the presence of FGF2, have been also reported to favor the formation of putative human PGCs in vitro [126]. In addition, silencing the *NANOS3* genes in human ESCs resulted in a marked reduction in the capability to give rise to PGC‐LCs [130]. These PGCLCs show some PGC markers, ongoing removal of parental imprinting, erasure of global DNA methylation, and histone modifications typical of mouse PGCs supporting the PGC identity. Furthermore, expression of DAZ family genes with spontaneous differentiation in human ESCs apparently induced 1 % of haploid‐like cells with some meiotic markers [131]. Some reports show that human ESCs and iPSCs express a panel of PGC markers such as AP, SSEA4, OCT4, NANOG, STELLAR (stella‐related), and BLIMP1, DAZ, DAZL, NANOS1, NANOS3 in some but not all ESC lines, and c‐KIT, but not SSEA1, CXCR4, and VASA or synaptonemal complex protein 1 and 3 (SCP1 and SCP3) markers of pre‐ and meiotic germ cells. On the other hand, ESCs and iPSCs express some markers that human PGCs do not, such as SSEA3, tumor rejection antigen 1–60, 1–81 (TRA1‐60, TRA1‐81), and SOX2 [46].

Cynomolgus monkey ESCs show NANOS, SSEA1, OCT‐4, and VASA and PIWIL1 expression during spontaneous differentiation which results in embryoid body formation [132, 133]. The addition of BMP4 to differentiating ESCs increased the expression of SCP1, a meiotic marker [133]. After 8 days of differentiation, LIF addition induced dome‐shaped germ cell colonies as indicated by the intense expression of AP activity. These cells also demonstrate high‐level expression of the germ cell markers VASA, OCT‐4, and BLIMP‐1, and show SSEA‐1 expression [134]. Additionally, in common marmoset ESCs, upon non‐directed differentiation, the cells expressed the germ cell markers VASA, BOULE, germ cell nuclear factor (GCNF) and SCP3 [112]. Pig EpiSCs in response to BMP4 induce VASA and DAZL‐positive PGC‐LC [104].

While mouse ESCs are difficult to differentiate directly into PGC‐LCs, human and especially primate pluripotent cells seem to have a tendency to differentiate spontaneously into PGC‐LCs. This might be because non‐rodent PSCs are in a ‘primed state’ and possibly with some PGC precursors already in the heterogeneous population in the colonies. However, mouse EpiSCs, which are called ‘primed’ state, differentiate into PGC‐LCs with very low efficiency. This suggests that the mouse ‘primed’ EpiSCs and ‘primed’ human ESCs have a different ability for PGC differentiation. On the other hand, mouse EpiLCs, which are differentiated from mouse naive ESCs, have a high ability to become PGCs and functional germ cells after in vivo transplantation. The human spontaneous PGC differentiation protocol in vitro is not as efficient as the defined mouse protocol. To improve the efficiency of human PGC‐LC induction in vitro, they may require progression towards a more competent state as a starting point.

## Perspective

In 1859, Charles Darwin concludes that “community of embryonic structure reveals community of descent”. He suggested that embryonic resemblance could be a strong remark for the genetic connectedness of different animal groups [135].

To understand the biological fundamental process of organisms, we have to choose the organism as an experimental model dependent on the purpose or practical technical reasons. To apply the results or interpretation which is from one organism to another organism has to be judged carefully. When we focus on early mammalian development, differences between rodents and other species are evident. Germ cells are the only cells able to give rise to the next generation, and they are set aside during early development. An investigation on this key event is informative on the continuity of life. The mechanisms for establishing a germline are diverse. Even in mammals, pluripotency and germ cell specification are quite different at molecular and cellular morphological levels.

A non‐negligible observation here is that rats and mice share an unusual method of early formation of egg cylinder epiblast and form unique characteristics of ESCs/iPSCs. In contrast, other mammals show epiblast delamination as a simple flattened embryonic disc and flattened, primed colonies of ESCs/iPSCs. The derivation and maintenance of mESCs/iPSCs with the same condition of human ESCs or human ESCs/iPSCs with the same condition of mouse ESCs/iPSCs without additional treatment has not been demonstrated. It might suggest that it may not be the quality or timing of the embryo, but that the naive and primed pluripotency are representative of pluripotent states of rodents and non‐rodents, respectively. The validity of the ground state hypothesis for other mammals besides rodents is open to further investigation. Germ cells and in vitro PSCs share some features in terms of molecular regulatory mechanism. To understand embryogenesis and in vitro pluripotency regulation of mammals comparatively would give some clues on the mechanism of PGC specification and development. With the emergence of new genome editing tools, there are opportunities for broader insights and deeper knowledge on early embryogenesis and stem cell science across different species.

## Acknowledgments

This work was supported by grants from BIRAX Regenerative Medicine Initiative. W.W.C.T. is funded by Croucher‐Cambridge International Scholarship, jointly supported by Croucher Foundation and The Cambridge Commonwealth, European & International Trust. We would like to thank Dr. Roopsha Sengupta, Dr. Kei Miyamoto, Dr. Carlos le Sage, Dr. Toshihiro Kobayashi for critical reading of the manuscript.

### Conflict of interest

Naoko Irie, Walfred W.C. Tang and M. Azim Surani declare that they have no conflict of interest.

### Informed consent

All procedures followed were in accordance with the ethical standards of the responsible committee on human experimentation (institutional and national) and with the Helsinki Declaration of 1964 and its later amendments. Informed consent was obtained from all patients for being included in the study.

### Human/Animal Rights

This article does not contain any studies with human or animal subjects performed by any of the authors.
